# Meningeal Relapse of Nodular Lymphocyte Predominant Hodgkin Lymphoma Transformed to T-Cell/Histiocyte-Rich Large B-Cell Lymphoma: A Case Report

**DOI:** 10.3389/fonc.2020.01745

**Published:** 2020-09-14

**Authors:** Paolo Salvioni Chiabotti, Bettina Bisig, Anne Cairoli, Steven D. Hajdu, Pierre-Yves Lovey, Dina Milowich, Sonia Ziadi, Renaud Du Pasquier, Jean-Philippe Brouland, Laurence de Leval

**Affiliations:** ^1^Department of Clinical Neurosciences, CHUV University Hospital and University of Lausanne, Lausanne, Switzerland; ^2^Department of Laboratory Medicine and Pathology, Institute of Pathology, CHUV University Hospital and University of Lausanne, Lausanne, Switzerland; ^3^Department of Oncology, CHUV University Hospital and University of Lausanne, Lausanne, Switzerland; ^4^Department of Diagnostic and Interventional Radiology, Lausanne University Hospital and University of Lausanne, Lausanne, Switzerland; ^5^Department of Medicine, Service of Hematology, Sion, Switzerland

**Keywords:** Central nervous system, nodular lymphocyte predominant Hodgkin lymphoma, case report, transformation, cerebrospinal fluid, autopsy, T-cell/histiocyte-rich Large B-cell lymphoma

## Abstract

Central nervous system involvement in Hodgkin lymphoma is extremely rare, especially in nodular lymphocyte predominant Hodgkin lymphoma (NLPHL), which usually carries a favorable prognosis. Here we report a case of a young patient with NLPHL, who developed a progressive and fatal neurological deterioration requiring a very extensive work-up including two biopsies to obtain the diagnosis of T-cell/histiocyte-rich large B-cell lymphoma like transformation. This report, which includes post-mortem analysis, highlights the correlations between clinical, radiological, and biological data but also the difficulties encountered in reaching the correct diagnosis.

## Introduction

Central nervous system (CNS) lymphoma is very often a diagnostic challenge and has been relatively frequently reported in systemic non-Hodgkin lymphoma (NHL) but very rarely in Hodgkin lymphoma (HL) (1–3). The anatomical sites affected can be diverse, including isolated leptomeningeal involvement (4). Nodular lymphocyte predominant Hodgkin lymphoma (NLPHL) is a rare disease accounting for approximately 5–10% of HL (5) and affects predominantly males at a median age of 30–35 years. It typically presents with stage I or II disease carrying a very favorable prognosis with a 10-years overall survival of greater than 90%. However, approximately 20% of the patients with advanced-stage NLPHL have an inferior survival, similar to classical HL (6), and tend to relapse or transform to an aggressive B-cell lymphoma which may resemble T-cell/histiocyte-rich large B-cell lymphoma (THRLBCL), or more usual diffuse large B-cell lymphoma (7). Here, we report a case of a patient with NLPHL relapsing with meningeal involvement, manifested by progressive and fatal neurological deterioration, and with THRLBCL-like transformation.

## Case Description

**FIGURE 1:**
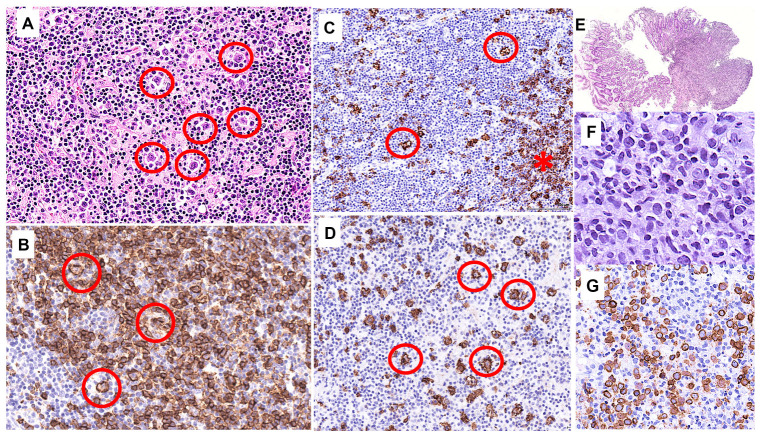
Axillary lymph node biopsy (April 2017; **A–D**) and gastric biopsy (February 2019; **E–G**). **(A)** High power view of the lymph node shows a few large LP cells scattered within a background of small lymphocytes and histiocytes. **(B)** CD20 immunostaining in a pattern A (classical) nodule highlighting many small B cells and scattered large LP cells (red circles). **(C)** CD20 immunostaining in pattern C [nodular with many extranodular lymphocyte predominant (LP) cells] showing the edge of a nodule (asterisk), and scattered large LP cells (red circles) outside of the nodule **(D)** CD20 immunostaining showing large CD20+ atypical cells (red circles) arranged in large nodules with essentially no small B cells in the background (pattern D: T-cell-rich nodular). **(E)** Low-power view of the gastric biopsies showing a dense and destructive cellular infiltrate. **(F)** The infiltrate comprises lymphoid cells including large atypical elements. **(G)** CD79a highlights the rather numerous large atypical B cells.

A previously healthy 31-year-old man first presented in 2017 with fever, asthenia, mild self-resolving headache, and pancytopenia. PET-CT showed enlargement of multiple peripheral, thoracic, and abdominal lymph nodes, several hypermetabolic foci in the liver, splenomegaly and focal pancreatic uptake [maximal standardized uptake value (SUV) 23.8, at hepatic hilum], and an axillary lymph node biopsy (Figures 1A–D) showed NLPHL comprising different immunoarchitectural patterns, mostly C [nodular with many extranodular lymphocyte predominant (LP) cells] and D (T-cell-rich nodular), with a minor component of classical A pattern (B-cell rich nodular). The LP cells were BCL2- CD10- lymphoid and the Epstein-Barr virus (EBV) was negative by *in situ* hybridization. The bone marrow biopsy was negative. The disease was therefore staged IV-B, with an International Prognostic Score (IPS) score of 2/7. Six cycles of R-CHOP (Rituximab-Cyclophosphamide Doxorubicin Vincristine Prednisone) followed by two cycles of Rituximab were administered between May and July 2017. An interim PET-CT showed good partial response, while thoraco-abdominal CT at the end of treatment showed a persisting right axillary lymph node (1.8 cm) and slight splenomegaly, without focal lesions. In the absence of an end-of-treatment PET-CT, the patient was followed clinically. In February 2019 he reported abdominal pain, vomiting, and weight loss. A bulging of the gastric wall was observed through gastroscopy, and biopsies (Figures 1E–G) revealed an atypical destructive lymphoid infiltrate in the mucosa, containing a moderate number of large CD79a+, BCL2+, CD10-lymphoid cells, surrounded by abundant T cells, interpreted as relapse with THRLBCL-like lymphoma. A PET-CT showed hypermetabolic abdominal lymph nodes and several hypermetabolic foci with a maximum SUV of approximatively 16, localizing to the stomach, small bowel, pancreas, and spleen. Salvage chemotherapy [three cycles of R-ICE (Ifosfamide Carboplatinum Etoposide)] induced a complete metabolic response, followed by autologous stem cell transplantation in May 2019 with no major complications.

**FIGURE 2:**
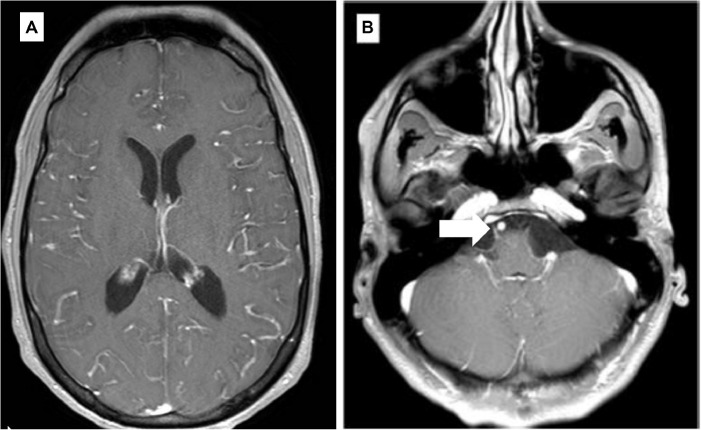
Selected axial T1 weighted images from brain MRI with contrast **(A,B)**. **(A)** Diffuse gyriform leptomeningeal enhancement on both cerebral hemispheres. **(B)** Enhancement of the cisternal portion of the right 6th cranial nerve (arrow).

**FIGURE 3:**
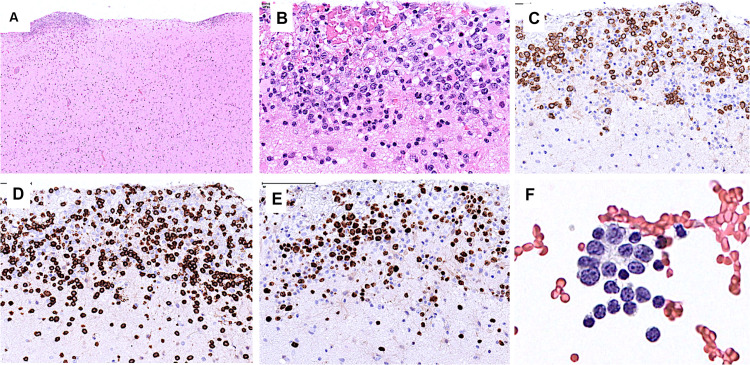
Brain biopsy **(A–E)** and CSF **(F)**. **(A)** Low power view of the brain cortical biopsy showing cellular deposits on the meningeal surface. **(B)** The cellular foci comprise many large atypical cells. **(C)** The latter are positive for CD79a. **(D)** CD3 stains many admixed small T cells. **(E)** Ki67 stains the nuclei of most large cells. **(F)** CSF contains a few large atypical cells and many small lymphocytes.

**FIGURE 4:**
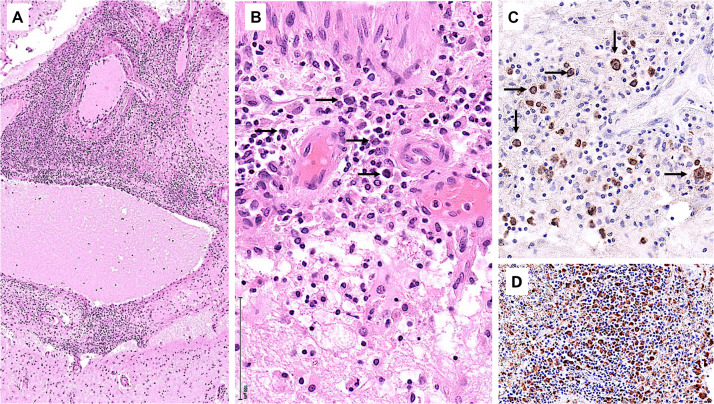
Brain autopsy. **(A)** Low power view of leptomeningeal spaces enlarged by a dense cellular infiltrate. **(B)** The infiltrate comprises scattered large lymphoid cells (arrows), numerous small lymphocytes, and histiocytes. **(C)** CD79a highlights the large atypical B cells (arrows). **(D)** CD68 underscores the histiocyte-rich background.

Starting July 2019, he presented with a progressive headache, neck stiffness, fever, and asthenia. The initial work-up showed intracranial hypertension (up to 50 cm H2O), lymphocytic meningitis (pleocytosis up to 220 cells/μl, between 80 and 95% lymphocytes), elevated cerebrospinal fluid (CSF) lactate levels (up to 9 mmol/l), very low CSF-to-serum glucose levels (ratio down to 0.12), increased CSF protein (up to 12 g/l), as well as diffuse leptomeningeal and cranial nerve enhancement, without any parenchymal lesion on magnetic resonance imaging (Figure 2). Flow cytometry analyses on four consecutive lumbar punctures found lymphocytic pleocytosis, with a T-cell immunophenotype (both CD4+ and CD8+), and PCR analyses showed polyclonal TRG gene rearrangements, while cytopathological analyses concluded to small lymphocytes without atypia and couldn’t disclose malignant cells. Despite extensive search for infectious agents, no definitive diagnosis could be established. Furthermore, no hypermetabolic foci were found on PET-CT, nor abnormalities on slit lamp examination, nor mass lesion on thoraco-abdominal CT. Empirical administration of broad-spectrum antibiotics (including antituberculotic agents), antifungals, IV immunoglobulins, and IV steroids (up to 12 mg/d dexamethasone) were unsuccessful. Clinical worsening was characterized by development of tetra-appendicular ataxia and delirium, with persistent low-grade fever. After a first inconclusive right frontal cerebro-meningeal biopsy (showing only reactive gliosis), a second one, performed 1 month later in the same region (Figure 3), found on the meningeal surface a tiny focus of large atypical lymphoid cells with a CD20-/(+), CD79a+, CD10+, BCL2+, BCL6+, MYC+, MUM1-, CD30- immunophenotype, admixed with fibrin and a moderate amount of small CD3+ reactive T cells. The Ki67 proliferation fraction was >90%. It was concluded to a focus of large B-cell lymphoma. Fluorescent *in situ* hybridization (FISH) studies found no rearrangement of *BCL2, BCL6*, or *MYC* genes while cytological examination of the CSF obtained at the same time showed marked small-cell lymphocytosis with scattered large atypical cells. B-cell clonality studies (IGH, IGK, and IGL gene rearrangements) were non-contributive on the brain biopsy but demonstrated monoclonal peaks in the CSF. The patient received three injections of high-dose IV methylprednisolone and high dose methotrexate (3 g/m2), but his clinical condition did not improve and he finally died 3 months after the initial neurological complaints. A brain autopsy was performed. Histologically, the main finding was a diffuse cellular infiltrate in the meningeal spaces, predominantly in the sulci, featuring THRLBCL-like characteristics (Figure 4). The infiltrate consisted of small CD3+ lymphocytes, histiocytes, and scattered large hyperchromatic lymphoid cells that were positive for CD79a and CD19. *In situ* hybridization for EBV was negative. No sheets of large cells nor necrosis was observed.

## Discussion

The clinical course of this young patient, who presented with advanced-stage NLPHL treated with R-CHOP frontline therapy, was characterized by initial abdominal recurrence with visceral involvement in the form of a THRLBCL-like transformation and finally meningeal relapse. The incidence of transformation to aggressive B-cell lymphoma in NLPHL is estimated in the range of 7 to 10% at 10 years (8, 9), and the reported median time to transformation is several years (8–10). Advanced stage disease seems to be associated with a higher risk of transformation (8, 11), and there have been reports suggesting that most of the stage III/IV patients harbor disease already transformed at the time of presentation (12). This may have been the case also in our patient where the maximum SUV observed on the first PET-CT was above the usual values for NLPHL (13). Splenic involvement may have been another risk factor of transformation (9). While the variant histologic patterns of NLPHL have been found to correlate with advanced disease at initial diagnosis, a higher risk of recurrence and poor clinical outcomes after stage-adapted first-line treatment (14), they have not been formally established as risk for transformation.

The most unusual aspect of this case was the occurrence of a second relapse with isolated CNS involvement. CNS involvement in HL is extremely rare at diagnosis and in patients with relapsed or refractory disease. In a recent study, Cheah et al. screened the clinical data of more than 30,000 HL patients and identified only 21 cases with CNS involvement, corresponding to an incidence <0.1% (3). Two retrospective series have reported a total of 37 HL patients with CNS involvement (2, 3). With the exception of one case of NLPHL who had CNS involvement at the time of the diagnosis, the other 36 cases were classical HL with roughly half of them presenting CNS involvement at the time of primary diagnosis, and the other half presenting CNS relapse. Most cases were associated to parenchymal lesions identified by brain imaging, and less than 25% of the cases had leptomeningeal involvement only. A variety of unspecific neurological symptoms have been described, including weakness, headache, pain and sensory changes, altered mental status, seizures, or manifesting with lymphocytic meningitis without visible lesions detectable by imaging. Atypical cells were identified in the CSF in a minority of cases, while increased protein levels in the CSF was a common finding.

The patient reported here is the first well-documented case of meningeal involvement in relapsed/transformed NLPHL. This diagnosis has been excessively difficult requiring two biopsies, and thus highlighting several key points that might have delayed the diagnosis and subsequently treatment. In addition to the very low likelihood of CNS involvement in HL, the systemic disease was in remission. Metabolic and imaging studies, which are usually sensitive in detecting relapsed HL, failed to reveal any abnormal cerebral uptake. Therefore, both the clinical picture and CSF characteristics suggested an unknown infectious or reactive meningeal process. The paucity of neoplastic cells and lack of parenchymal brain involvement were further confounding factors. Post-mortem analysis of the brain demonstrated widespread THRLBCL-like histology involving solely the meninges, with no parenchymal infiltrates, correlating very well with the clinical, biological, and radiological findings. This case therefore illustrates that all patients with NLPHL presenting with neurological symptoms should undergo thorough neurologic investigation including detailed neuroimaging, CSF analysis with molecular testing for clonality, and possibly brain biopsy. After exclusion of alternative diagnoses, lymphoma should still be considered even if all the aforementioned ancillary examinations come back negative.

## Ethics Statement

Written informed consent was obtained from the participant for the publication of any potentially identifiable images or data included in this article.

## Author Contributions

PSC: study conception and design, acquisition of data, analysis and interpretation, and writing of the manuscript. BB: acquisition and analysis of data and critical revision of the manuscript for important intellectual content. AC: acquisition of data and critical revision of the manuscript for important intellectual content. SH: acquisition of data, neuroimaging figure preparation, and critical revision of the manuscript for important intellectual content. P-YL: acquisition of data and critical revision of the manuscript for important intellectual content. DM: acquisition of data and critical revision of the manuscript for important intellectual content. SZ: acquisition of data and critical revision of the manuscript for important intellectual content. RDP: study conception and design, data analysis and interpretation, and critical revision of the manuscript for important intellectual content. J-PB: acquisition of data, analysis and interpretation, and critical revision of the manuscript for important intellectual content. LdL: study conception and design, acquisition of data, analysis, and interpretation, writing of the manuscript. All authors contributed to the article and approved the submitted version.

## Conflict of Interest

The authors declare that the research was conducted in the absence of any commercial or financial relationships that could be construed as a potential conflict of interest.
